# Screening of Antioxidant Activity of *Gentian Lutea* Root and Its Application in Oil-in-Water Emulsions

**DOI:** 10.3390/antiox3020455

**Published:** 2014-06-19

**Authors:** Nurul Aini Mohd Azman, Francisco Segovia, Xavier Martínez-Farré, Emilio Gil, María Pilar Almajano

**Affiliations:** 1 Chemical Engineering Department, Technical University of Catalonia, Avda. Diagonal 647, 08028 Barcelona, Spain; E-Mails: ainiazman@gmail.com (N.A.M.A.); segoviafj@gmail.com (F.S.); 2 Chemical and Natural Resources Engineering Faculty, University Malaysia Pahang, LebuhrayaTunRazak, Pahang 26300, Malaysia; 3 Agro-Food Engineering and Biotechnology Department, EsteveTerradas, 8, 08860 Castelldefels, Spain; E-Mails: Xavier.Martinez-Farre@upc.edu (X.M.-F.); Emilio.Gil@upc.edu (E.G.)

**Keywords:** *Gentiana Lutea*, lipid oxidation, antioxidant, HPLC

## Abstract

*Gentiana Lutea* root (*G. Lutea*) is a medicinal herb, traditionally used as a bitter tonic in gastrointestinal ailments for improving the digestive system. The active principles of *G. Lutea* were found to be secoiridoid bitter compounds as well as many other active compounds causing the pharmacological effects. No study to date has yet determined the potential of *G. Lutea* antioxidant activity on lipid oxidation. Thus, the aim of this study was to evaluate the effects of an extract of *G. Lutea* on lipid oxidation during storage of an emulsion. *G. Lutea* extracts showed excellent antioxidant activity measured by DPPH scavenging assay and Trolox equivalent antioxidant capacity (TEAC) assays. An amount of 0.5% w/w *G. Lutea* lyophilise was able to inhibit lipid oxidation throughout storage (*p* < 0.05). A mixture of *G. Lutea* with 0.1% (w/w) BSA showed a good synergic effect and better antioxidant activity in the emulsion. Quantitative results of HPLC showed that *G. Lutea* contained secoiridoid-glycosides (gentiopiocroside and sweroside) and post column analysis displayed radical scavenging activity of *G. Lutea* extract towards the ABTS radical. The results from this study highlight the potential of *G. Lutea* as a food ingredient in the design of healthier food commodities.

## 1. Introduction

*Gentian Lutea* root (*G. Lutea*), also known as Yellow Gentian, has over 300 species and is widely distributed in North America, Europe, Asia and some parts of South America [1]. The plant root was traditionally use as a medicinal plant to stimulate appetite and improve digestion [2]. Furthermore, the root is also well known for its bitter properties due to the existence of secoiridoid-glycosides (e.g., swertiamarin, gentiopicroside, amarogentin and sweroside) [3]. Many researchers have discovered the benefits of bitter tasting secoiridoid-glycoside through extensive pharmacological studies. These constituents are claimed to have many biological effects such as anti-apoptotic [4], anti-cancer [5], anti-fungi [6], anti-bacterial [7], anti-inflammatory [8], and hepatoprotective [9]. It has been reported recently by Nastasijevic *et al*. [10] that the *G. Lutea* extracts have potential to inhibit myeloperoxidase (MPO) activity which contributes to many disorders such as cardiovascular, inflammatory, neurodegenerative and immune-mediated diseases.

However, not only are the secoiridoids relevant for the plant pharmacological action, there are many active compounds in *G. Lutea* that also have relevant effects such as iridoidloganic acid, xanthone (e.g., gentisin and isogentisin) and xanthone glycosides (gentioside and its isomer) [3]. Iridoidloganic acid has shown potent activity as an anti-inflammatory and a number of studies demonstrated that xanthones and its derivatives have wide-ranging biological activities such as anti-inflammatory, anti-hepatotoxic, anti-tumor and anti-microbial [11]. Furthermore, xanthone, and its derivatives, are phenolic compounds with antioxidant properties which have attracted much attention recently [12]. There are few reports that have investigated the radical scavenging activities of secoiridoid-glycosides using DPPH assay [7,13], although some authors have measured total antioxidant capacity of *G. Lutea* but without sufficient assessment of individual antioxidants [10,14]. However, the observation of antioxidant activity of *G. Lutea* extract towards lipid oxidation has not been fully determined.

Lipid oxidation in high fat-containing food is a major cause of shelf life deterioration such as in meat products and emulsions. The oxidation process causes unsavoury alteration of flavor, texture, shelf life, appearance, and nutritional qualities [15].

Oil-in-water emulsion (o/w), is a food model which is highly susceptible to oxidation, besides it has also become one of the effective models to evaluate the antioxidant activity of natural plants towards lipids [16]. The first phase of lipid oxidation starts with the formation of unstable free radicals and hydroperoxides with further decomposition to secondary products like ketones, aldehydes, alcohols and acids [17]. The formation of peroxides in primary oxidation can be measured using peroxide value (PV) assay. Secondary oxidation can be measured by thiobarbituric acid reacting substances (TBARS), specifically aldehydes, and also leads to unpleasant taste, aroma and quality traits of the products.

Thus, our goal was to evaluate the potential antioxidant activity of *G. Lutea* by different methods (1) *in vitro* with such radicals as ABTS^•+^, DPPH and enzymatic activity and (2) in o/w emulsion.

## 2. Experimental Section

### 2.1. Plant Material

Commercially dried *G. Lutea* was kindly supplied by Manatial de la Salut (Barcelona, Spain), a registered herbal company. Reagents used were: thiobarbituric acid, 1,1-diphenyl-2-picrylhydrazyl (DPPH), Folin-Ciocalteu reagent, methanol, hydrogen chloride, aluminium oxide, ferrous chloride, anhydrous sodium carbonate, ethanol 96%, Phosphate Buffer Solution (PBS) and ammonium thiocyanate from Panreac (Barcelona, Spain). Gallic acid, 2,2′-azino-bis (3-ethylbenzothiazoline)-6-sulfonic acid diammonium salt (ABTS), (±)-6-hydroxy-2,5,7,8-tetramethylchromane-2–carboxylic acid (Trolox), NBT (nitrobluetetrazolium), Bovine Serum Albumin (BSA), Xanthine and Xanthine-oxidase from Sigma-Aldrich (Gillingham, UK).

### 2.2. Extraction of G. Lutea

Dried roots of *G. Lutea* were finely ground using a standard kitchen food processor. Ground *G. Lutea* (5 g) was extracted in two ways; (1) with 50:50 (v/v) methanol:water and (2) with water, always in the ratio 1:10 (w/v). The extraction was performed at 4 ± 1 °C for 24 h, in the dark with constant stirring. The extract solutions of *G. Lutea* were recovered by filtration using Whatman Filter paper, 0.45 μm. Part of the supernatant was taken for subsequent use to determine the antiradical capacity. The volume of the remaining supernatant was measured and the excess methanol was removed under vacuum using a rotary evaporator (BUCHI RE111, Postfachi, Switzerland) and kept frozen at −80 °C for 24 h. All extracts were dried in a freeze dryer (Unicryo MC2L −60 °C, Martinsried, Germany) under vacuum conditions at −60 °C for 3 days to remove moisture. Finally, *G. Lutea* lyophilize (freeze dried) were weighed to determine the concentration recovered (g/L) and the extraction yield (%) as Zhang *et al*. [18]. Samples were then weighed and kept protected from light in a desiccator until use.

### 2.3. Determination of the Total Phenolic Content (TPC)

The Folin-Ciocalteu method was used to determine the total phenolic content (TPC) as reported by Santas *et al*. [19]. The sample was diluted 1:25 (v/v) in order to be in the range of absorbance. The final concentration (v/v) for the mixture was; sample 7.7%, Folin reagent 4% and saturated sodium carbonate solution 30.8%. The mixture was finally diluted with Mili Q water, shaken and incubated in the dark for 1 h. Absorbance at 765 nm was measured using a microplate reader (Fluostar Omega, BMG Labtech, Ortenberg, Germany) against water as a blank. Gallic acid was used to prepare a standard calibration, and the results were expressed as mg of Gallic acid equivalents/g dry weight (mg GAE/g DW).

### 2.4. Determination of Free Radical Scavenging Activity Assays

#### 2.4.1. TEAC Assay

The antioxidant capacities of *G. Lutea* were measured by using a modified TEAC assay, which was performed as described by Miller *et al*. [20]. The TEAC assay was based on the reduction of the ABTS^•+^ radical cation by the antioxidants present in the samples. ABTS^•+^ radical cation (7 mM, final concentration) was dissolved before adding potassium sulphate (2.45 mM, final concentration) and allowing the mixture to stand in the dark up to 16 h. Phosphate Buffer Solution (PBS, 10 mM) with the ABTS^•+^ radical cation was incubated at room temperature for 30 min before used. Then, the mixture of the ABTS^•+^ radical cation was adjusted to an absorbance of 0.73 ± 0.2 nm, using a microplate reader (Fluostar Omega, BMG Labtech, Ortenberg, Germany). The TEAC values for the different concentrations of each compound were interpolated from the trolox calibration curve and expressed as milligrams of trolox equivalent per gram of dry weight sample (mg TE/g DW sample).

#### 2.4.2. DPPH Assay

The effect of the extracts on the scavenging of DPPH radical was determined according to the method adapted from Madhujith *et al*. [21] with slight modifications. The sample was diluted 1:20 (v/v) and DPPH radical in methanol (5.07 mM) was made for the study. Then, the sample (10% v/v) and DPPH solution (90% v/v) were added to the well of the microplate. The absorbance was measured at 517 nm over every 15 min for 75 min. The results were expressed as mg TE/g DW sample.

#### 2.4.3. Superoxide Activity Xanthine/Xanthine Oxidase (X/XO)

The method was based on the developed method of Valentao *et al*. [22] and modified for application in microplates by Lopez *et al*. [23]. All test samples were dissolved in a 50 mM phosphate buffer to simulate the environment in which the reaction occurs in the body. The sample was mixed with 145 μM of a solution of xanthine, 50 μM of a solution of NBT and incubated in 37 °C. The sample extract was diluted from 1:10 to 1:100 (v/v) for the study. Finally, 0.29 U/mL of enzyme xanthine oxidase solution was added and the absorbance was recorded at 560 nm every 2 min. The value of IC_50_ was calculated to determine the inhibition rate of *G. Lutea* in the reaction.

### 2.5. Determination of Antioxidant Activity in o/w Emulsion

#### 2.5.1. Removal of Tocopherols from Sunflower Oil

Alumina was placed in an oven at 200 °C for 24 h, and then removed and allowed to cool in a desiccator until it reached room temperature. Sunflower oil triacylglycerol was passed twice through the alumina in a column to remove the tocopherols as described by Yoshida *et al*. [24]. Finally, the filtered oil was stored at −80 °C until use.

#### 2.5.2. Preparation of Emulsion

Oil in water emulsion was prepared by dissolving Tween-20 (1%, final concentration) in Milli Q water and adding oil (10%, final concentration). To form an emulsion, the oil was added drop wise to the solution of Tween-20 and water, which was kept cold, and sonication process was continued for 5 min. All samples were redissolved in ethanol-50% (v/v) to obtain the final concentration in the emulsion. The final samples were prepared either (i) control (no addition); (ii) 0.35% (w/w) Trolox (positive control); (iii) 0.1% (w/w) BSA; (iv) 0.5% (w/w) lyophilise *G. Lutea*; (v) 0.5% (w/w) lyophilise *G. Lutea* mixed with 0.1% (w/w) BSA; (vi) 0.2% (w/w) lyophilise *G. Lutea* and (vii) 0.2% (w/w) lyophilise *G. Lutea* mixed with 0.1% (w/w) BSA. The emulsion for each sample was prepared in quadruplicate, obtaining a total of 28 samples and stored in the dark and allowed to oxidize at 37 °C. The pH of the samples was measured four times for each sample (pH meter GLP21, Crison Instruments, Barcelona, Spain) as a parameter to investigate its correlation with PV.

#### 2.5.3. Determination of Peroxide Value (PV)

The primary oxidation products were measured using peroxide value (PV) according to the thiocyanate method of the Association of Official Analytical Chemists (AOAC) 8195 [25]. Ferrous chloride solution was prepared in hydrochloric acid (1 M) with the addition of iron chloride (II) (2 mM, final concentration). Ammonium thiocyanate solution was prepared in water (2 mM, final concentration). The assay was performed with a drop of emulsion in the range from 0.007 to 0.01 g, diluted with ethanol. From this solution the required amount of sample, varying according to the degree of oxidation, was taken in a cuvette and ethanol (96%) was added. Ferrous chloride and ammonium thiocyanate solutions were added, each in a proportion of 1.875% (v/v), final concentration. The absorbance was measured spectrophotometrically at λ = 500 nm. The results are expressed as meq hydroperoxides/kg of emulsion.

#### 2.5.4. Determination of Secondary Oxidation by Thiobarbituric Acid Reactive Substances (TBARS)

The TBARS method was adapted from Gallego *et al*. [26]. The TBARS reagent was prepared (15% w/v trichloroacetic acid, 0.375% w/v thiobarbituric acid and hydrochloric acid 2.1% v/v). One mL of each emulsion was taken and the TBARS reagent was added in the ratio 1:5 (v/v). Immediately the samples were added to an ultrasonic bath (5 min) and after immersing in a water bath preheated to 95 °C (20 min) the samples were centrifuged and the absorbance of the supernatant was measured at λ= 531 nm. The results are expressed as mg malondialdehyde (MDA)/kg of emulsion.

### 2.6. Statistical Analysis

Statistical analysis was performed using a one-way analysis of variance ANOVA using Minitab 16 software program (Minitab, Inc., Paris, France). When a statistically significant difference was found, Tukey’s tests were performed and the statistical significance was set at *p* < 0.05. The results were presented as mean values (*n* ≥ 3).

### 2.7. HPLC and Post-Column HPLC-ABTS^•+^ Radical Scavenging Method

The method for identification of peaks with antioxidant activity was that used by Koleva *et al*. [27] with some modifications. The instrument was a Waters 2695 separations module (Meadows Instrumentation Inc., Bristol, USA) system with a photodiode array detector Waters 996 (Meadows Instrumentation Inc., Bristol, USA). The column used was a Kinetex C18 100A, (100 × 4.6 mm, Phenomenex, Torrence, CA, USA). Solvents used for separation were 0.1% acetic acid in water (v/v) (eluent A) and 0.1% acetic acid in methanol (v/v) (eluent B). The gradient used was isocratic, 75% A. The flow rate was 0.6 mL/min. Detection wavelength was 230 nm (to see the peaks) and 734 nm (to see the ABTS radical). The sample injection volume was 10 μL. The chromatographic peaks of gentiopicroside and sweroside were confirmed by comparing their retention times and diode array spectra with that of their reference standards. The pump for ABTS post-column injection was a Merk-Hitachi HPLC gradient pump (Model L-6200, Hitachi High Technologies America Inc., Schaumburg, Illinois, IL, USA) with a 0.2 mL/min flow; ABTS concentration was of 0.03% (w/v).

## 3. Results and Discussion

### 3.1. Analysis of Total Polyphenols and Free Radical Activity Assays

On average, from 5 g of dried *G. Lutea* extracted with aqueous methanol 50:50 (v/v) and water alone, it was possible to recover 1.5 ± 0.05 g and 1.0 ± 0.04 g of lyophilised, respectively. The concentration recovered was proportional to the extraction yield shown in Table 1. Previous studies reported that gentiopicroside compound, an active compound that signifies the main bitter principle in *G. Lutea* was still preserved at almost 83.5% after drying [28].

**Table 1:** Extraction yield, total phenolic content (TPC), 1,1-diphenyl-2-picrylhydrazyl (DPPH), Trolox equivalent capacity assay (TEAC) and enzymatic activity of *G. Lutea*.

Activity *G. Lutea*	Extraction Solvent	
	H_2_O	50:50 MeOH:H_2_O
Extraction yield (%)	20.00 ± 0.9	29.10 ± 0.3
Total phenolic content (g GAE/g DW)	3.79 ± 1.7	12.03 ± 1.8
DPPH (μmol of TE/g DW)	12.34 ± 1.5	15.89 ± 0.5
TEAC (μmol of TE/g DW)	33.28 ± 1.5	48.90 ± 1.8
Superoxide activity (mg/mL)	30.00 ± 2.8	23.21 ± 2.8

Mean value *n* = 3 and the standard deviation for each assay is less than 5%. Gallic Acid Equivalent (GAE), Trolox Equivalent (TE), Dry Weight (DW).

The concentration of total polyphenols and the value of antioxidant activity assays were determined and the results are shown in Table 1. The extract of *G. Lutea* in methanol-50% showed higher phenolic content and antioxidant activity than the water extract. The total phenolic content of *G. Lutea* extracts allowed the estimation of all phenolic acids, flavonoids, anthocyanins, nonflavonoids and many classes of polyphenol compounds present in the samples. On the other hand, Nastasijevic *et al*. determined the total polyphenol content of *G. Lutea* in water extract as being slightly higher compared to different concentrations of aqueous ethanol and methanol extracts [10].

Water extracts of *G. Lutea* showed the lowest activity in free DPPH^•^ scavenging activity compared to methanol-50% extract, similar to previous research from Kintzios *et al*. [14]. It is not the first time that the antioxidant activity of *G. Lutea* by DPPH method has been carried out. However, variation in results may be due to the plant age, solvent, method and system used throughout the experiment [10,14,29].

For the TEAC assay, the finding was consistent with the DPPH method where the aqueous methanol extract showed higher activity than the water extract. TEAC assay indicated the extract potency used as a source of antioxidants based on the ability of the antioxidant compound to scavenge the long-life radical cation ABTS^•+^. In the results shown in Table 1 it can be appreciated that the methanol-50% extract has a higher capacity to scavenge ABTS^•+^ radicals and consequently shows a higher antioxidant activity than DPPH assay. To the best of our knowledge, this is the first report of the antioxidant activity of extracts from *G. Lutea* roots assessed using the TEAC methods.

Some of the previous reports showed that the antioxidant activity of plant extracts correlates with the phenolic content [30] and the yield of phenolic components from herbs is higher with methanol-50% rather than with water as extract. The mixtures of alcohol and water have been more efficient in extracting compounds and give a better yield than the corresponding mono-component solvent system. Xanthones such as isogentisin and gentisin and its derivatives are one of main sources of phenolic compounds in *G. Lutea* and are expected to be more soluble in aqueous alcohol. A study showed good correlation between phenolic content and antioxidant activity [31] whereas another found no correlation [32].

In the present work, an effective antioxidant activity in *G. Lutea* was found. Methanol-50% extract exhibited O_2_^•−^ scavenging activity, measured using the X/XO system (Table 1), with an IC_50_ at 23.21 ± 2.8 mg/mL. Water extract of *G. Lutea* showed lower scavenging activity than methanol aqueous extract, with IC_50_ = 30.00 ± 2.8 mg/mL. These results are consistent with Kusar *et al*. [29], who demonstrated the effect of superoxide activity of *G. Lutea* leaf and root in methanol extracts, with IC_50_ inhibition value of 11.1 mg/mL and 8.2 mg/mL, respectively. Kusar and co-workers’ findings were accomplished by X/XO reaction mixture with DEPMO-OOH scavenger that transformed the reaction to a stable radical measured by electro spin resonance (ESR). Valentao *et al*. [22] observed the phenolic acids (*p*-coumaric acid, ferulic acid, sinapic acid and kaempferol) exhibited superoxide scavenger activity and an inhibitory effect on XO. Considering the results obtained from TPC assay, it may be anticipated that *G. Lutea* extract has antioxidant activity achieved by the scavenging of superoxide radical and XO inhibition.

### 3.2. Antioxidant Effect in Stored o/w Emulsion

Many strategies on laboratory scale have been developed to improve the stability of shelf life in food models including adding a minimum amount of natural plants to delay the oxidation rate. The effect of *G. Lutea* on inhibiting lipid oxidation in oil-in-water (o/w) emulsion as a food model has not been described. In this study it also has been carried out to determine the synergic effect of *G. Lutea* with BSA in o/w emulsions. The oxidation in o/w emulsions was measured in two stages of oxidation; primary oxidation product (Peroxide Value) and secondary oxidation products (TBARS). In addition the change in pH was monitored, since pH tends to fall during oxidation.

#### 3.2.1. Evolution of Peroxide Value (PV)

**Figure 1:**
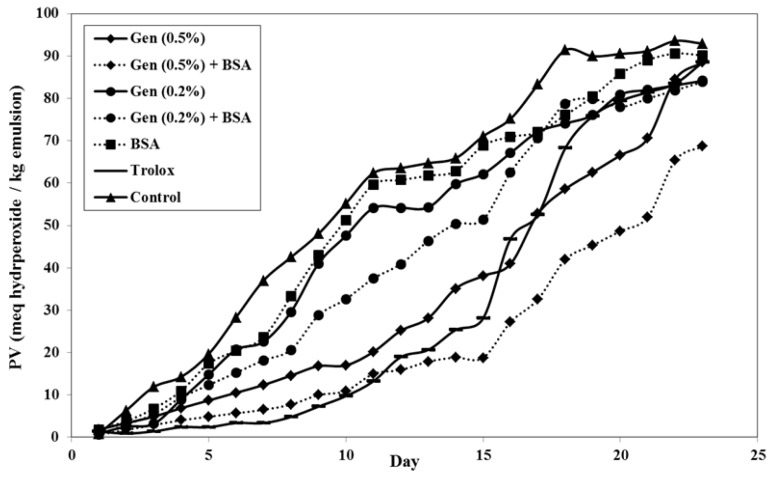
Change of peroxide value over time stored at 37 °C (each value is expressed as mean (*n* = 3)).

Figure 1 shows the evolution of PV *vs*. time. The control (without extract added) showed the highest oxidation throughout the storage time followed by the emulsion with only BSA (0.1%). The sample containing Trolox (0.35%, positive control) and the samples containing extracts, were not oxidized during the first 10 days. They show significant difference from the control (*p* < 0.05). The time required for the emulsions to reach a peroxide value of 10 meq hydroperoxides/kg of emulsion was determined as a standard to measure the stability of emulsion. The limits of fat product (animal, plant and anhydrous) margarine and fat preparation were set <10 meq hydroperoxides/kg as a guarantee of the product quality [33]. When the peroxide value of the sample is measured as greater than 15 meq hydroperoxide/kg, the sample is considered rancid, which may alter the color, taste and nutritional quality due to the deterioration of the lipid. The control was the first sample to reach 10 meq hydroperoxides/kg of emulsion which occurred rapidly in two days. The emulsion with BSA exhibited a similar deterioration rate to the control, revealing that BSA, in this concentration of 0.1%, does not provide any antioxidant effect in the emulsions. Positive control samples (Trolox) showed good antioxidant effect over 11 days and begin to oxidize rapidly after 15 days, reaching 88 meq hydroperoxides/kg emulsion on the final days of the experiment.

Adding 0.2% *G. Lutea* to the emulsion, with or without adding BSA, did not result in any relevant effect towards oxidation in the first stage (PV <10 meq hydroperoxides/kgin time <3 days). There is a significant difference between 0.2% antioxidant sample with BSA and in its absence (*p* < 0.05). The sample with 0.5% *G. Lutea* showed antioxidant activity towards lipid degradation first at 15 days and gradually oxidized after 15 days (*p* < 0.05). Finally, the sample with *G. Lutea* 0.5% and BSA 0.1% displayed the lowest PV, with significant differences with the other samples throughout storage time (*p* < 0.05); it took almost 10 days to reach above 10 meq hydroperoxides/kg of emulsion.

Almajano *et al*. [34] reported that some antioxidant compounds such as EGCG and caffeic acid mixed with BSA cause a marked increase of the antioxidant activity in an emulsion. Since BSA is known to be surface active [35], the increase of antioxidant activity in emulsions containing a mixture of antioxidant and BSA could be due to BSA binding with the antioxidant and transporting it to the oil water interface, where it is highly effective in reducing the rate of oxidation. The authors also stated that the antioxidant molecule had bound to the BSA protein, proved by TEAC assay, and showed a progressive increase in the radical scavenging ABTS^•+^ with the storage time over several days [34]. The mixture of 0.1% BSA and 0.5% *G. Lutea* in emulsion exhibit the lowest oxidation rate compared to all samples shown in this experiment. These results showed for the first time the important effect of *G. Lutea* extract on lipid oxidation with synergic effect to BSA tested in an o/w emulsion. This concentration of 0.5%, demonstrated the best antioxidant effect throughout the storage period.

#### 3.2.2. Evolution of pH over Time

**Figure 2:**
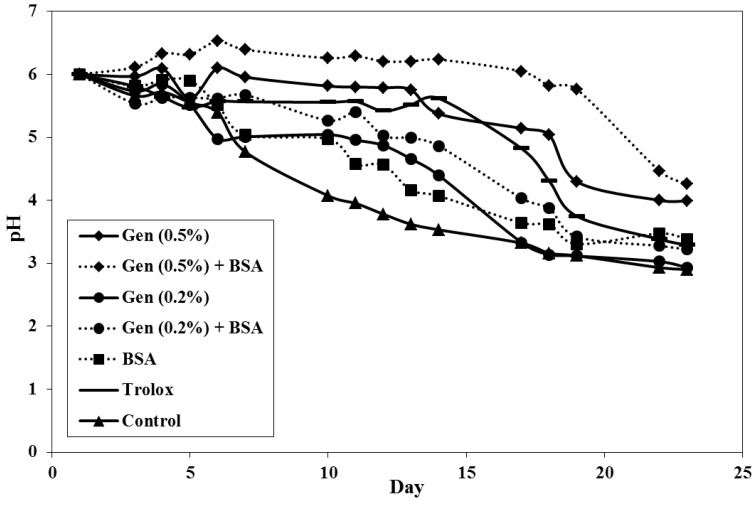
Change of pH over time, stored at 37 °C (each value is expressed as mean (*n* = 3)).

Since decomposition of hydroperoxide measured in PV assay is acidic, the pH change in the sample is considered inversely proportional to the PV. Thus, the pH measured is a parameter of which its correlation with PV can be investigated. Antioxidant activity in food models is less effective under low pH conditions. However, some antioxidant compounds such as carnosic acid and carnosol (found in rosemary) have been reported to have high antioxidant activity at lower pH, which is at pH 4–5 [36]. Figure 2 shows the changes of pH value on emulsions over 23 days storage. Overall, the decrease in pH value throughout storage was of a similar order with increased primary oxidation measured in the PV assay. These results agreed with the studies done by Frankel *et al*. [16]. They found that the lipid oxidation in emulsion is slower at higher pH, and decreased when oxidation is accelerated. All samples started nearly at neutral pH and 0.5% *G. Lutea* with 0.1% BSA showed the highest value throughout storage. Similar to PV, the pH of the sample with 0.2% *G. Lutea* with or without BSA showed higher pH than control but the value was not significant throughout storage (*p* > 0.05). The pH of 0.5% *G. Lutea* with BSA and 0.5% *G. Lutea* alone, showed significant differences compared to all samples during storage period (*p* < 0.05). The behavior of pH of the sample with 0.5% *G. Lutea* with BSA was stable until 19 days before it started to decrease.

Skowyra *et al*. [37] demonstrated that the pH and PV have the best correlation with *R*^2^ = 0.9648. Our results are in agreement with them and it can be described that the antioxidant activity in o/w emulsion which is stable at pH 6 showed an inverse relationship at a lower PV value. Some authors also reported a similar agreement of pH change which was inversely proportional to the lipid oxidation [26,38,39]. Meanwhile, Mancuso *et al*. [40,41] suggested the initial oxidation of emulsion depended on pH, by varying the effect of emulsifier. The authors observed a higher oxidation rate occurring at pH 7 rather than pH 3 o/w emulsion. Results may be due to the iron solubility increasing at low pH and allowing iron to be partitioned into the continuous phase, whereas insoluble iron at high pH may precipitate onto the emulsion droplet surface resulting in an increase in the lipid oxidation.

#### 3.2.3. Evolution of Thiobarbituric Acid Reactive Substances (TBARS)

**Figure 3:**
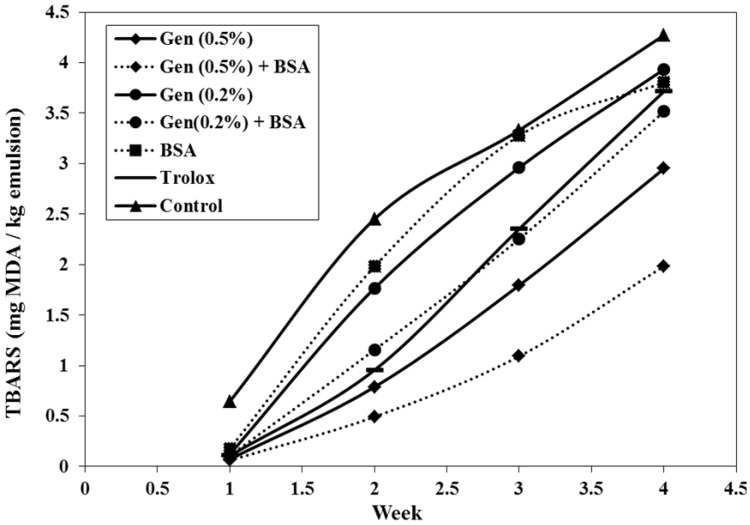
Change of Thiobarbituric Acid Reactive Substances (TBARS) over time, stored at 37 °C (each value is expressed as mean (*n* = 3)).

One of the compounds produced from secondary oxidation in lipids is MDA (malondialdehyde) which can be measured by the TBARS method. The secondary lipid oxidation is responsible for the alteration of flavor, rancid odor and the undesirable taste in foods [42]. Secondary oxidation products were monitored by TBARS assay and are shown in Figure 3. Similar to PV, the control had the most rapid increase in TBARS followed by the BSA sample. TBARS values for samples treated with 0.5% gentian powder, with and without BSA, experienced below 1.2 mg MDA/kg sample over the first 21 days and showed prominently lower than positive control up to 4 weeks (*p* < 0.05). The sample with 0.2% of *G. Lutea* alone does not display significant delay in lipid oxidation (*p* > 0.05), meanwhile 0.2% *G. Lutea* with BSA and positive control showed significant different during 20 days (*p* ≤ 0.05). From the TBARS results exhibited, the synergic effect between both the concentration of *G. Lutea* and BSA in the emulsion during the storage time is demonstrated and the samples with both (gentian and BSA) show the lowest oxidation rate compared to all samples.

After 23 days, all samples are oxidized with above 1.2 mg malondialdehyde/kg sample even though *G. Lutea* with the BSA mixture showed minimum rise and had the best antioxidant effect in the emulsion. This behavior is not new. It has been previously reported that artificial antioxidants such as Trolox, epigallocatechingallate (EGCG), caffeic acid are more stable in emulsions during storage in the presence of BSA than in its absence [34].

*G. Lutea* has compounds which of family of iridoids, scoiridoids and xanthones [3]. It could be developed as antioxidant agent and radical scavengers and may contribute to the decrease of lipid oxidation [43]. The water soluble antioxidant molecular structures differ in the number of phenolic hydroxyl groups, their location and the carboxylic acid group [34]. Thus, the range of structure presented may allow any possible interaction with the BSA to be detected.

Many findings determined such amount of natural plants work as antioxidant efficiently; depended on variation of system tested and plant preparation. However, the more important is to identify the minimum concentration required to reduce lipid oxidation significantly throughout storage time. Adding minimum amount of natural plant not only can delay lipid oxidation, but also to avoid some changes on sensorial quality and flavoured. Analysis on our data showed for *G. Lutea* required as minimum 0.5% (w/w) would be beneficial for reducing the velocity of lipid oxidation significantly in both primary and secondary oxidation in emulsion system (*p* < 0.05). Adding also 0.1% (w/w) of BSA gave better effect of the antioxidant activity towards emulsion, compare to sample with *G. Lutea* alone. The study confirmed the potential of edible *G. Lutea* to prevent oxidation of emulsion.

### 3.3. HPLC Analysis of G. Lutea and the Total Antioxidant Activity Based on Post-Column On-Line Coupling ABTS^•+^

The HPLC analysis is targeted to identifysecoiridoid-glycoside, the bitter constituent occurred in the extract shown in (a) and (b). Results in Table 2 presentsecoiridoid glycoside; gentiopicroside, swerosideand amarogentin were the important compounds in the *G. Lutea*. The highest content of secoiridoidin *G. Lutea* extract in methanol-50% weregentiopicroside (1805 ± 62 mg/L extract) and the amount of sweroside found was 72 ± 4 mg/L extract. It was not possible to identifyamarogentin in the extract, meanwhile only low traces of amarogentinwere found in various commercial *G. Lutea* (less than 0.09%) [3]. Carnat *et al*. [28] also reported that amarogentin can only be found in some fresh root of *G. Lutea*. There are comprehensive studies measuring the active compound in *G. Lutea* using bioassay-guided fractionation such as HPLC [3], CapilaryElectrophoris [44] and Thin Layer Chromatography [45]. From the literature study, there is constituent of secoiridoidthat has not been identified in this study (swertiamarin)are believed to be existed in the extract [2]. This is most likely due to the objective of the method was not optimize the quantification of the traces but to measure the antioxidant activity of each compound in the *G. Lutea* extract.

**Table 2:** Amount of secoiridoid-glycoside quantified by HPLC.

Sample	Concentration (mg/L)
Gentiopocroside	1805 ± 62
Sweroside	72 ± 4
Amarogentin	n.d

n.d = not detected.

Aberham *et al*. [3] analyzed the active compound of 12 commercial samples of *G. Lutea* root. They found that swertiamarin was shown to have consistent occurrence between 0.21% and 0.45% and gentiopicroside was the most dominant compound in the sample up to 9.53%. Meanwhile, Ando *et al*. [46] reported that gentiopicroside was not detected from the fresh roots of 3-year-old *G. Lutea*. In contrast, Hayashi *et al*. [47] described that one year root contains high amounts of gentiopicroside and amarogentin and decreases over 5 years.

The Gentianaceae family is well known for its intensive bitter root used as a tonic for the digestive system with many pharmacological benefits. There are other plants such as *Swertiachirayita* [48] and *Lonicera japonica* [49] that also possess similar compounds of secoiridoid-glycosides (swertiamarin and sweroside).

Investigation of the main individual compounds in *G. Lutea* root was previously developed and optimized by Aberham *et al*. [2,3]. Furthermore, a substantial number of studies have demonstrated the effect of the secoiridoid group on the scavenging function to generate free radicals [7,13,48]. Wei *et al*. [13] reported that five secoiridoids, including gentiopicroside, sweroside, swertiamarin and sweroside, did not show any scavenging ability towards free radicals by *in vitro* DPPH assay. However, taking into account their report, it was desirable to explore more individual extracted compounds by isolating the compounds followed by a biochemical assay, such as ABTS radical, to measure their activity. Online post-column methods are very dependable because they combine systems for investigating different features of the sample simultaneously. Our initial observation of using *in vitro* ABTS assay showed that gentiopicroside and sweroside displayed no scavenging activity towards ABTS radicals (data not shown) while the activity of these compounds is similar towards DPPH radicals. However, our finding showed an activity of amarogentin analyzed by ABTS *in-vitro* assay (644.5 ± 17.5 mg eq TE/L sample). However, the amarogentin was not identified in the extract, as discussed above. This is in contrast with Phoboo *et al*. [48] who observed no scavenging activity of amarogentin towards DPPH radical.

**Figure 4:**
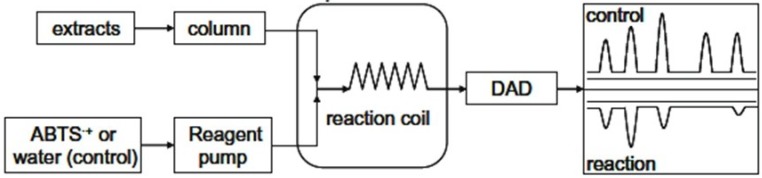
Scheme of HPLC-ABTS for screening of antioxidant compounds in *G. Lutea* root extract.

**Figure 5:**
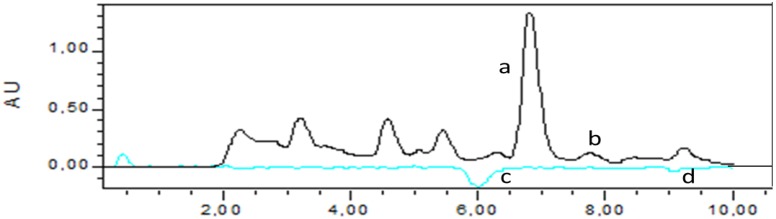
Chromatogram of *G. Lutea* root extract obtained direct and from the post-column HPLC-ABTS^•+^ radical scavenging method. (**a**) Gentiopicroside; (**b**) Sweroside; (**c**) antiradical activity by unknown compound; 31.33 ± 1.16 mg GAE/L and (**d**) antiradical activity by unknown compound; 8.30 ± 0.12 mg GAE/L.

The HPLC separated analytes reacted with ABTS radical post column (see Figure 4) and the reduction was detected as a negative peak at 734 nm. In Figure 5, the chromatographic analysis showed gentiopicroside (a) and sweroside (b) detected in *G. Lutea* extract and unknown compounds, (c) and (d), detected as negative peaks using the ABTS radical assay, which indicated that these components had free radical scavenging activity. The result of antioxidant response peaks (negative peak) of the *G. Lutea* compounds, expressed as mg galic acid equivalent (GAE)/L extract, indicates their relative contribution to the antioxidant activity of the extract with concentration taken into account. Analysis of *G. Lutea* extract of total antioxidant activity had been reported several times mainly measured by DPPH *in vitro* assay [10,14]. Even though we were unable to identify the compound relevant to the scavenging activity in the post-column ABTS assay, the results showed that the antiradical capacity of *G. Lutea* is not related to gentiopicroside and sweroside. There are many other compounds that maybe related to the scavenging activity in the extract such as xanthone-glycosides.

Nevertheless, our finding showed that the 0.5% *G. Lutea* extracts is able to delay the process of oxidation and give better storage stability as emulsion. However, this activity is not due to the bitter compounds (gentiopicroside amarogentin and sweroside) presented in our extract. Studies (from the revised literature) proved that xanthone compounds such as gentioside, gentisin and isogentisin found in *G. Lutea* have antiradical activity even though their constituents possess remarkable activity in pharmacological study

## 4. Conclusions

*G. Lutea* has valuable pharmacological properties due to the bitter properties of its secoiridoid-glycosides; their extraction using methanol-water mixture was better than water alone. *G. Lutea* extract showed excellent antioxidant activity in aqueous methanol measured by DPPH scavenging activity assay and Trolox equivalent capacity assay (TEAC) methods (15.89 and 48.90 μmol of TE/g DW, respectively). *G. Lutea* lyophilize can be applied as antioxidants in oil-in-water emulsions. 0.2% (w/w) of lyophilize. *G. Lutea* does not inhibit lipid oxidation significantly. An amount of 0.5% (w/w) *G. Lutea* lyophilise exhibited antioxidant activity towards primary and secondary oxidation in an o/w emulsion. Adding 0.1% (w/w) BSA with *G. Lutea* in an emulsion showed a synergic effect and better activity in delaying lipid oxidation.

Gentiopicroside and sweroside found in HPLC analysis do not show any antiradical capacity in *G. Lutea* aqueous methanol extract. However, total antiradical capacities shown in post-column measurements presented activities of 31.33 ± 1.16 mg GAE/L and 8.30 ± 0.12 mg GAE/L towards the ABTS free radical. This study confirmed that *G. Lutea* roots as a source of edible natural antioxidants have potential to be used by the food industry.
